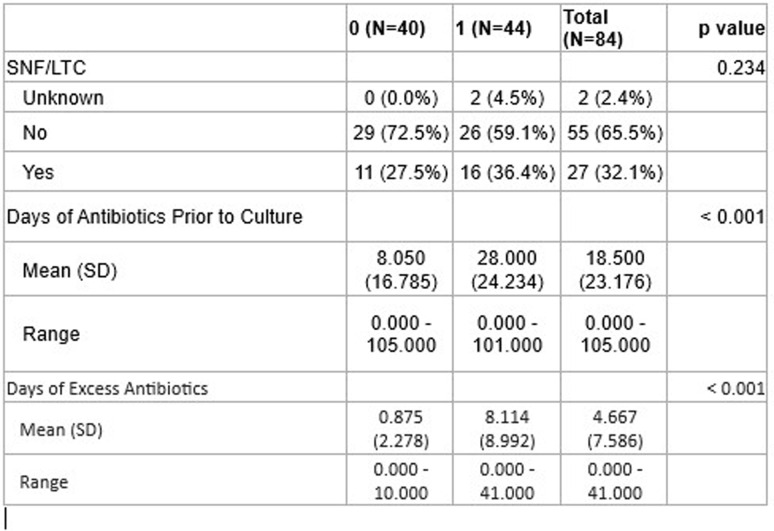# 37 Parent and Caregiver Preferences Towards Antibiotic Allergy Delabeling in Children

**DOI:** 10.1017/ash.2026.10478

**Published:** 2026-06-23

**Authors:** Jamil Muqtadir, Maya Polashenski, Mackenzie Kross, Emily Nguyen, German Vargas-Cuebas, Moein Mozafari, Yoon Kwak, Jason Bennett, Jennifer Gutowski, Melissa Bronstein, Michele Kolb, Francois Lebreton, Emil Lesho

**Affiliations:** 1 Rochester Regional Health; 2 WRAIR | DCB | MRSN

## Abstract

**Background:** Candida auris (CA) poses an urgent public health threat due to its multidrug-resistance, associated mortality, and ability to persist in the inanimate healthcare environment. We sought to ascertain factors associated with a prolonged multi-facility outbreak that was not amenable to previously successful containment measures involving Clade 1. **Methods:** Contact tracing and testing, environmental sampling, cleaning observations with intensified cleaning, and whole genome sequencing were performed. Additionally, dry hydrogen peroxide units (Synexis DHP® Overland Park, KS) were installed, damaged furniture was replaced, and availability of dedicated equipment was increased. Standard urine culturing protocols were changed to better detect and identify yeast. Exposed patients that remained uncolonized were compared to exposed colonized patients in a nested case control exploratory analysis using multivariable logistic regression with excess antibiotic days as the primary exposure in the adjusted model. Covariates were selected based on prior understanding of the pathogen, biological plausibility, and univariable analyses to explore crude associations. We created one primary adjusted model, with additional sensitivity analyses conducted to avoid overfitting due to the small sample size and potential confounding. Model discrimination was evaluated using the area under the curve. **Results:** All isolates in the initial clusters were Clade III and highly genetically related to each other. Later a small cluster of highly related Clade 1 emerged. Patient variables and descriptive statistics appear in Table 1. After adjusting for age, sex, number of rooms the patient occupied, and Charlson comorbidity score, each additional day of unjustified antibiotics prior to culture was associated with a 63% increase in the odds of CA positivity (OR=1.63 per day, 95% CI: 1.22-2.53, p=0.007). Number of patient rooms occupied prior to culture was also strongly associated with positivity (OR=1.83 per additional room, 95% CI 1.37-2.82, p=0.0007). Discussion We applied the same containment measures that had previously succeeded, along with those described above, but were unable to control this outbreak. At the time of this submission, CA continued to be detected sporadically in clinical cultures, supporting our hypothesis that clade III is more difficult to contain than Clade I. This report is notable because it confirms our earlier findings of CA emergence linked solely to excess antibiotic exposure, using a more robust study design. As excess antibiotic days was independently associated with CA positivity, it underscores the importance of aggressive antimicrobial stewardship to prevent emergence. Once CA establishes itself, containment may be impossible.

**Figure:**
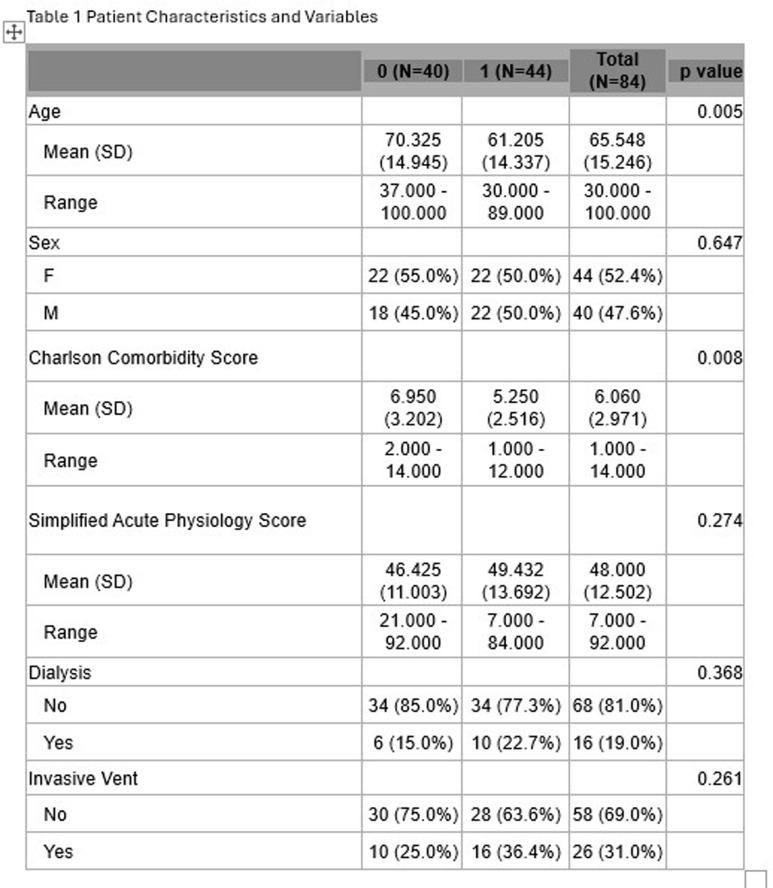

**Figure:**
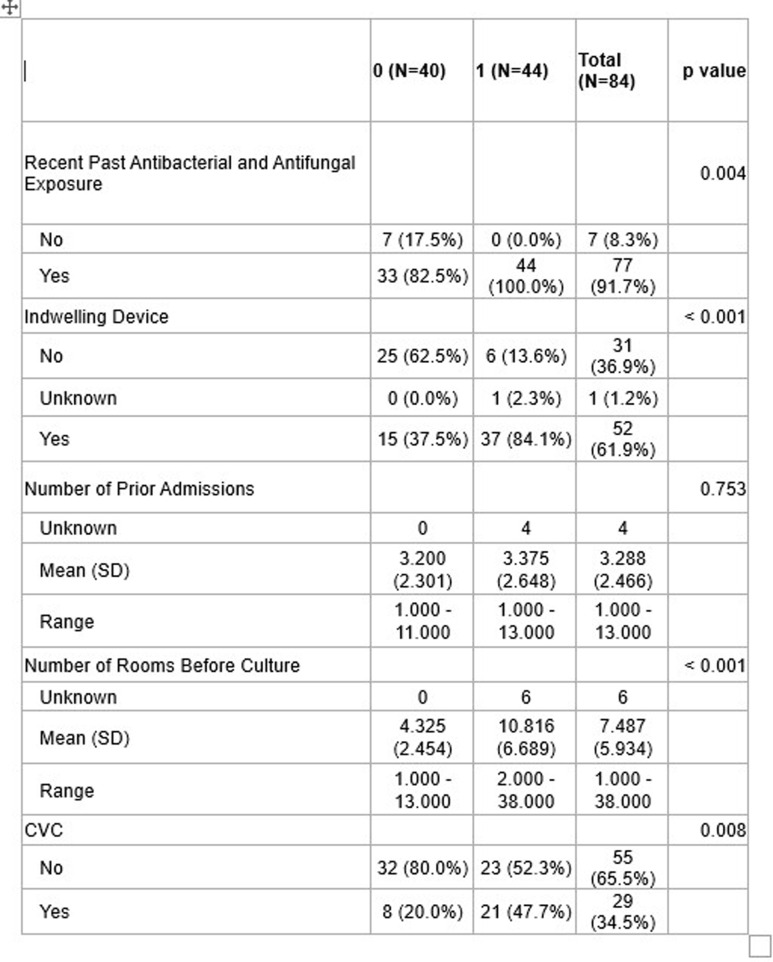

**Figure:**